# Remember oligodendrocytes: Uncovering their overlooked role in Alzheimer’s disease

**DOI:** 10.1371/journal.pbio.3002798

**Published:** 2024-09-12

**Authors:** Lena Spieth, Mikael Simons

**Affiliations:** 1 Institute of Neuronal Cell Biology, Technical University Munich, Munich, Germany; 2 German Center for Neurodegenerative Diseases (DZNE), Munich, Germany; 3 Munich Cluster of Systems Neurology (SyNergy), Munich, Germany; 4 Institute for Stroke and Dementia Research, University Hospital of Munich, LMU Munich, Munich, Germany

## Abstract

Our understanding of Alzheimer’s disease has evolved from focusing solely on neurons to recognizing the role of glia. This Primer discusses a recent study in PLOS Biology that showed that oligodendrocytes are an important source of Aβ that impairs neuronal function.

## Teaser

Our understanding of Alzheimer’s disease (AD) has evolved from focusing solely on neurons to recognizing the role of glia. A recent study in *PLOS Biology* revealed that oligodendrocytes are an important source of Aβ that impairs neuronal function.

The amyloid hypothesis has served as a conceptual framework for Alzheimer’s disease (AD) research, describing how amyloid peptides initiate a pathological cascade leading to neurodegeneration and dementia. Initially, this concept had a neuron-centric view, where neurons were both the producers and victims of toxic amyloid aggregates. However, it has since been significantly modified by integrating the cellular phases into the pathophysiological cascade [1]. Genome-wide association studies in AD marked a turning point, expanding the focus beyond the neuron-centric amyloid cascade hypothesis to include the contributions of microglia. Yet, the roles of other glial cell types have been largely overlooked until recently.

Two studies, one in *PLOS Biology* and the other in *Nature Neuroscience*, now highlight the role of oligodendrocytes in the disease [2,3]. The starting point for both Rajani and colleagues [2] and Sasmita and colleagues [3] was the analysis of RNA sequencing datasets [4], which revealed that oligodendrocytes express all essential genes required for Aβ production, including APP, BACE1, and components of gamma secretase. Surprisingly, the levels of these genes rank highest in oligodendrocytes compared to other cell types. Proteomic data from isolated mouse brain cells confirmed that oligodendrocytes contain high levels of these proteins [5]. Further validation using RNAscope in situ hybridization on postmortem AD and control brains showed that approximately 80% of oligodendrocytes express both APP and BACE1, indicating their capability to produce Aβ.

These results align with a recently published study using freshly obtained human brain biopsy samples, which detected an increase in a set of genes responsible for regulating Aβ production not just in excitatory neurons but also in oligodendrocytes in the initial phases of AD [6]. To determine the contribution of oligodendrocytes to amyloid plaque load, both Rajani and colleagues [2] and Sasmita and colleagues [3] used an AD mouse model that expresses a humanized/mutated APP gene under its native regulatory elements and identified oligodendrocytes as contributors to Aβ accumulation. Targeted deletion of β-secretase, BACE1, in oligodendrocytes led to a roughly 30% reduction in total amyloid plaque load, including the soluble Aβ peptide fraction. This oligodendrocyte-derived contribution to Aβ was most significant in regions with a high density of oligodendrocytes, such as the white matter, where they accounted for up to about 50% of soluble Aβ peptides. However, both studies showed that deleting BACE1 specifically in neurons nearly eliminated amyloid plaque formation, highlighting the critical role of neurons and not oligodendrocytes in the generation of Aβ amyloid plaques.

**Fig 1:**
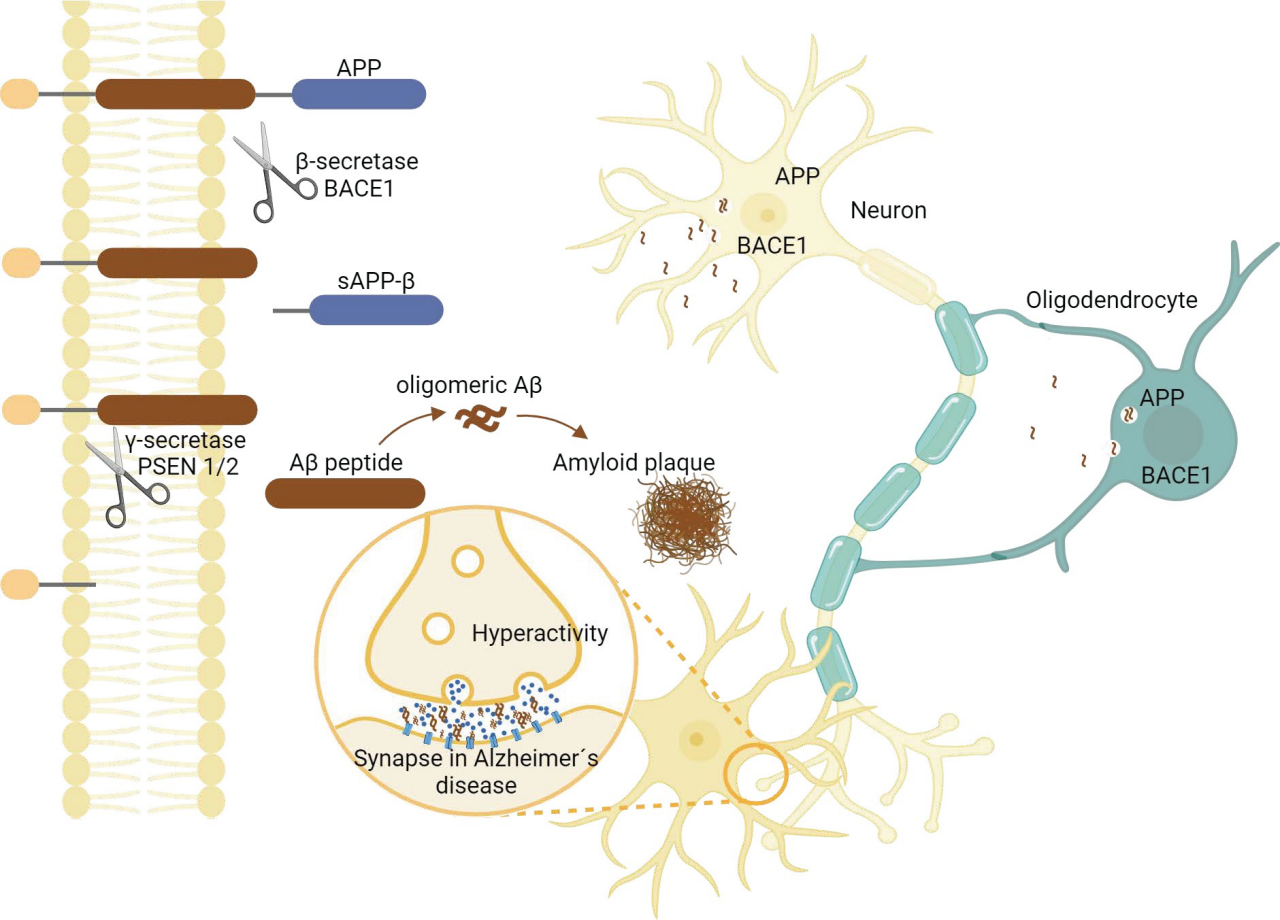
Amyloidogenic processing of the amyloid precursor protein (APP) in oligodendrocytes and its effects on neuronal activity. The transmembrane protein APP is sequentially cleaved by β-secretase (BACE1) and γ-secretase (PSEN1/2) in oligodendrocytes leading to the particular high Aβ42/40 ratios. The Aβ peptides released have the ability to aggregate into oligomers, which can lead to synaptic dysfunction accompanied by neuronal hyperactivity (graphics created with biorender.com).

What role do Aβ peptides originating from oligodendrocytes play? Previous research has identified a mechanism in Aβ-related neuronal dysfunction, characterized by impaired synaptic function that results in neuronal hyperactivity, creating a vicious cycle that worsens the disease over time [7]. Thus, Rajani and colleagues [2] investigated the impact of BACE1 knockout in oligodendrocytes on neuronal dysfunction in vivo using high-density single-unit cortical recordings. They demonstrate that oligodendrocyte-specific BACE1 knockout effectively reversed the early abnormal neuronal hyperactivity observed in AppNL-G-F mice, which is dependent on soluble Aβ. These results indicate that suppressing Aβ production specifically in oligodendrocytes mitigates neuronal hyperactivity, raising the question about the underlying mechanism. To explore this, Rajani and colleagues [2] differentiated oligodendrocytes from human induced pluripotent stem cells (iPSCs) derived from both familial AD patients and healthy controls to determine which forms of Aβ these cells generate. They found that these oligodendrocytes produced Aβ with an elevated Aβ42/40 ratio, which exhibited a greater propensity to aggregate into oligomers and protofibrils compared to Aβ produced by neurons. Injecting Aβ-rich media from human oligodendrocytes into mouse brains increased neuronal firing, suggesting that oligodendrocyte-derived Aβ contributes to early AD-related neuronal dysfunction. Thus, this study proposes the intriguing idea that the downstream effects of Aβ may differ based on its cellular source, with Aβ derived from neurons primarily contributing to amyloid buildup and Aβ from oligodendrocytes predominantly affecting neuronal hyperactivity (Fig 1).

How can Aβ variants of varying lengths arise from distinct cell types? Previous research has shown that membrane thickness and organization affect the production of different Aβ species by γ-secretase [8]. Indeed, oligodendrocytes have a unique lipid composition characterized by elevated levels of cholesterol, glycolipids, and phospholipids rich in long-chain and saturated fatty acids [9]. This results in more densely packed and thicker membranes, potentially explaining the higher Aβ42/40 ratio observed in these cells. If oligodendrocytes produce substantial amounts of Aβ42, one might expect amyloid plaques to form in the white matter where these cells are predominantly located. However, amyloid plaques are much less frequent in the white matter compared to the grey matter. This prompts an intriguing question which specific cell types and states within the oligodendroglial lineage are responsible for generating Aβ. The lineage comprises oligodendrocyte progenitor cells (OPCs), pre-myelinating oligodendrocytes, and myelinating oligodendrocytes. In AD models, oligodendrocytes transition into what are known as disease-associated states [10–13], and, in addition, OPC proliferation increases, leading to their differentiation into premyelinating oligodendrocytes [14], which exhibit high biosynthetic activity. Therefore, it would be intriguing to investigate whether these AD-induced cell states, such as disease-associated oligodendrocytes and pre-myelinating oligodendrocytes that accumulate in the grey matter, contribute to Aβ generation. In conclusion, these 2 studies emphasize the involvement of oligodendrocytes in the advancement of AD. Moving forward, it will be crucial to deepen our understanding of how oligodendrocytes behave and function within the cellular phases of AD.

## Abbreviations

AD: Alzheimer’s disease
iPSC: induced pluripotent stem cell
OPC: oligodendrocyte progenitor cell
